# Fetal response to maternal hunger and satiation – novel finding from a qualitative descriptive study of maternal perception of fetal movements

**DOI:** 10.1186/1471-2393-14-288

**Published:** 2014-08-26

**Authors:** Billie Bradford, Robyn Maude

**Affiliations:** Graduate School of Nursing, Midwifery and Health, Victoria University of Wellington, P O Box 7625, 6242 Newtown Wellington, New Zealand

**Keywords:** Fetal development, Fetal movement, Maternal-fetal exchange, Hunger

## Abstract

**Background:**

Maternal perception of decreased fetal movements is a specific indicator of fetal compromise, notably in the context of poor fetal growth. There is currently no agreed numerical definition of decreased fetal movements, with the subjective perception of a decrease on the part of the mother being the most significant definition clinically. Both qualitative and quantitative aspects of fetal activity may be important in identifying the compromised fetus. Yet, how pregnant women perceive and describe fetal activity is under-investigated by qualitative means. The aim of this study was to explore normal fetal activity, through first-hand descriptive accounts by pregnant women.

**Methods:**

Using qualitative descriptive methodology, interviews were conducted with 19 low-risk women experiencing their first pregnancy, at two timepoints in their third trimester. Interview transcripts were later analysed using qualitative content analysis and patterns of fetal activity identified were then considered along-side the characteristics of the women and their birth outcomes.

**Results:**

This paper focuses on a novel finding; the description by pregnant women of fetal behaviour indicative of hunger and satiation. Full findings will be presented in later papers. Most participants (74% 14 of 19) indicated mealtimes were a time of increased fetal activity. Eight participants provided detailed descriptions of increased activity around meals, with seven (37% 7 of 19) of these specifying increased fetal activity prior to meals or in the context of their own hunger. These movements were interpreted as a fetal demand for food often prompting the mother to eat. Interestingly, the women who described increased fetal activity in the context of hunger subsequently gave birth to smaller infants (mean difference 364 gm) than those who did not describe a fetal response to hunger.

**Conclusions:**

Food seeking behaviour may have a pre-birth origin. Maternal-fetal interaction around mealtimes could constitute an endocrine mediated communication, in the interests of maintaining optimal intrauterine conditions. Further research is warranted to explore this phenomenon and the potential influence of feeding on the temporal organisation of fetal activity in relation to growth.

## Background

There has been very little improvement in stillbirth rates in high-income countries, in recent decades [1]. In more than 50% of cases of stillbirth, the pregnant woman has identified decreased fetal activity prior to diagnosis of fetal death [2]. An improved understanding of fetal behaviour in the context of adverse intra-uterine conditions is needed, so that new tools may be developed for identifying the fetus at risk [1]. Monitoring of fetal movements by pregnant women as a method of identifying fetal compromise was first proposed by Sadovsky and Yaffe in 1973 [3]. Despite promising results of fetal movement monitoring studies, none have identified a sufficiently robust method of screening for normal fetal activity to warrant large-scale introduction to antenatal care [4].

A barrier to harnessing the power of pregnant women’s ability to observe the wellbeing of their fetus is inadequate definitions of normal and pathologic levels of fetal activity [5, 6]. There is considerable variation between pregnant women in terms of numbers of fetal movements perceived, making preset numerical movement limits difficult to apply in screening tools. Maternal concerns about fetal activity, sometimes expressed in vague terms [7], may relate to a reduction in strength of movements [8], a change in movement pattern, or absence of fetal activity in situations the mother might normally expect movement [9, 10].

Qualitative aspects of fetal activity warrant consideration when investigating fetal behaviour in the context of adverse intrauterine conditions. Neurologists have increasingly emphasised quality of fetal movements over quantity as an indicator of normal neurological status [11, 12]. Maternal perception of fetal movements is a qualitative phenomenon. Yet, very little research has been conducted in exploring this phenomenon using qualitative methodologies [13, 14].

To understand the abnormal one must first understand the normal. The aim of this study was to explore normal fetal activity as described by pregnant women, in the hope that insights gained might contribute to better understanding of fetal behaviour in the context of compromise. This study raises the possibility of influence of maternal meals on patterns of fetal activity. In particular a complex pattern of activity indicative of fetal anticipation of a meal, hunger and satiation is outlined, which was associated in this cohort with smaller size at birth.

## Methods

This study employed a qualitative descriptive methodology [15]. Qualitative description is a straightforward qualitative approach used when a comprehensive summary of an event or phenomenon in the everyday terms of that event is required. It is an inductive approach, useful for situations where little is known about the subject or where a ‘patient’ voice is required [16].

In contrast to the more interpretive methods, larger sample sizes might be employed in Qualitative Description, in order to embrace variation. Penetration of the data is broad rather than deep so that greater transferability of the data to other clinical settings may be achieved. In interpreting the data, the researcher is exhorted to stick close to the data, emphasising description over interpretation. Inductive approaches are useful where little is known about a subject, as is the case with qualitative aspects of maternal perception of fetal movements. Qualitative methodologies are rarely employed in their purest form and researchers often work as *bricoleur*, fashioning the methodological approach from more than one discipline to suit the particular requirements of the investigation at hand [17]. Health care researchers are increasingly finding combining approaches useful for complex problems in health care [18]. Qualitative description is well suited for use in studies that combine qualitative and quantitative methods. The purpose when conducting a study using qualitative description is not to press-on to a univocal position but instead to embrace variation. When reporting on findings plain-language participant accounts are used and quasi-statistical analyses may be provided to summarise numerically the patterns emerging from the qualitative data [15].

It has been established that there is considerable variation between pregnant women in levels of fetal movement perceived [19–21]. For this reason a purposive sampling strategy was used whereby women in their first pregnancy were recruited. A sample size of 20 women was chosen, being small enough to manage the intensive process of qualitative data analysis and large enough that some variation might be captured. For this study, 21 low-risk women in their first pregnancy were recruited, via five community-based midwifery practices in a provincial city in the North Island of New Zealand. Eligible women had a singleton pregnancy, were under the care of a Lead Maternity Care (LMC) Midwife, and had good spoken English. Women, who had conditions warranting transfer of care to a specialist obstetrician at the time of enrolment, were not eligible. Two participants interviewed were later excluded as not meeting eligibility criteria (one due to parity, one due to high BMI at booking), leaving the final sample for analysis at 19.

Interviews were conducted at two time points in the third trimester: early (28-32 weeks) and late (37-41 weeks). Early first trimester interviews were conducted with 15 participants and term interview were conducted with 18 participants 13 were interviewed at both time points. This allowed for inter-pregnancy comparisons of fetal movements descriptions within the cohort and intra-pregnancy comparisons at each time point. Additional term participants were recruited in anticipation of a loss to follow-up of participants recruited for the first interview which only occurred in two cases. Recruitment was stopped when a sense of saturation was achieved in that no new data emerged from the final interviews.

Questions were both semi-structured and open-ended and primarily asked women to describe how their baby’s movements felt, patterns of movements over the day and any factors they noticed that appeared to increase or decrease their baby’s movements. An inductive approach to questioning was employed where responses were followed up with clarifying or expanding questions such as ‘what do you mean by…?’ or ‘how did that feel?’ in order to gain descriptions that were as detailed as possible. Interesting or unusual participant responses prompted additional questions to be added to the interview schedule, to be asked of later participants. Early third trimester interviews were conducted face-to-face in the setting of their usual antenatal care, and an audio recording made of the interview for later transcribing and analysis. Late third trimester interviews were conducted as described above, or in some instances by telephone. Analysis of the data involved qualitative content analysis [22] of the transcribed interviews, which were coded, organised into themes and then checked and rechecked against original recordings and transcripts. The final phase of data analysis involved consideration of the distribution of patterns emerging from the qualitative data according to participant characteristics and their birth outcomes. In particular patterns of movement reported were considered in relation to infant birthweight, as birthweight is an important retrospective indicator of fetal wellbeing. For these analyses the GROW programme (Gestation Related Optimal Weight), which adjusts for maternal height, weight, parity and ethnicity, as well as the sex and gestational age of the baby was used [23]. Some descriptive statistics were undertaken for this final part of the data analysis.

Ethics approval was obtained from the New Zealand Health and Disability Ethics Committee (Central Region), and locality approval for the various community sites was obtained prior to approaching participants. Written permission was obtained from the participants to record the interviews and also to access pregnancy, labour and birth records, in order to consider birth outcomes in relation to the descriptive accounts. This research has adhered to the guidelines for qualitative research review (RATS) [24]. Pseudonyms have been used to protect participants’ identity.

## Results

Maternal descriptions of fetal movements in this study changed over the course of the third trimester, with early third trimester movements being characterised by their great variation in both type and strength of movements. By term jerky or jolting movements had subsided and kicks were reduced in favour of rolling, stretching and pushing movements. Strength of movements was likely to be increased at term. Patterns of movement at both time points were commonly influenced by environmental factors such as; time of day, maternal position and ambulation, noises and maternal meals. One novel finding; a complex fetal response to maternal hunger, eating and satiation is discussed in this paper. Full findings will be presented in later papers.

During interviews, participants were asked whether there was a pattern to their baby’s movements and if so, to describe it. Mealtimes were identified by the majority of participants (73.6%, 14/19) as times when fetal activity was likely to be increased in some cases (6) no further detail was offered. In eight cases however participants explicitly recounted increased fetal movements interpreted by the mother as a response to hunger or eating. Of these eight, seven women (36.8% 7/19) described increased fetal activity in association with maternal hunger or the period prior to meals. Five of the women who had described increased fetal movement with hunger (26.3%) described a notable period of quieting following a meal which they interpreted as their baby being sated and content. The remaining two described a continuation of the increased activity after eating that had been noted with hunger. Just one woman described increased fetal activity after a meal, without having first described increased movements with hunger. Thus where fetal activity in relation to meals was described in detail by participants in this study, the predominant pattern was one of increased fetal activity prior to meals and decreased fetal activity following meals with a transition period during which some fetuses might continue their increased activity during and immediately after the meal and others might settle quickly into a quiescent period.

In describing changes in fetal activity around mealtimes participants commonly ‘voiced’ their baby in an attempt to convey the ‘attitude’ expressed by the fetal movements. The character of the increased activity prior to a meal was interpreted by the pregnant women as a fetal expression of anticipation of food, giving way to frustration when the meal was delayed. Whilst, the nature of the movements perceived following a meal were interpreted as indicating happiness or contentment. In this way fetal movements appeared to function as a form of communication between mother and baby, effectively prompting the mother to eat when she had gone for longer than usual without a meal and then indicating to the mother when the fetus was sated.

These changes in activity were outlined by participants primarily in response to the question “Is there anything that makes your baby move more or less than usual?” A typical example of increased fetal activity prior to meals is as below:

*Sometimes if I haven’t eaten for a while. It could just be me* [pause], *but you just feel that sometimes she’ll make her presence known and go “I need some food here” or something, well that’s what it feels like. And until you have something to eat and then she’ll settle down again. (Bridget, 37 weeks)*

Increasing fetal activity in advance of meals was further intensified where a meal was delayed. These descriptions were normally independent of sitting as the mother was often too busy to get a meal as in the example below;

*She gets very excited just before dinner time, like just before any meal time; and it’s not even when I'm cooking. Sometimes I can just walk past the room that has food in it and if I'm not eating within 20 minutes then she starts getting quite irate! Like I must have somehow triggered that I was out to eat and then when I didn't she was hugely disappointed. (Ruth, 39 weeks)*

Two women reported increased activity in association with hunger *and* the period following eating, describing the movements as changing qualitatively during the course of meal, as in the following example.

*So if I’m either hungry… I notice if I haven’t eaten, like that’s what happened this afternoon, she was not impressed. And she just gets really wriggly and really squirmy. And then after I’ve eaten, you know, so similar, although it doesn’t* [pauses] *it feels a lot more comfortable after I’ve eaten, but she goes through a similar thing where she gets quite active. (Roimata, 39 weeks).*

For the remaining five of the seven women who had described increased activity with hunger the post-prandial period was associated with a marked quieting of fetal activity.

**Figure 1:**
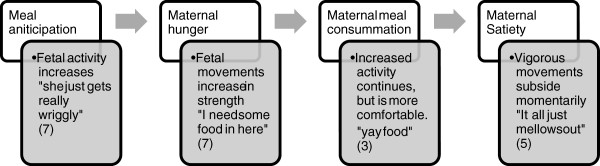
**Staged fetal responses to maternal hunger and eating, as described by participants.** Typical descriptions of fetal activity in relation to meal stage, with numbers of participants making a statement of this type.

*It seems to happen several times a day; like I suppose it is more around meal times, like before breakfast, she starts getting a bit excited, but yeah, afternoon tea time; any of those times as well, she's still, she goes: “Oh yeah, food’s on the way, yay.” … after a meal she's completely silent.... usually for, even up to an hour she’ll not, like I won't feel a single movement ‘cause it’s almost just like she's just chowing down and happy.* (Ruth, 39 weeks)Fetal activity as described by women in relation to hunger and eating demonstrated a developmental pattern over the course of a mealtime episode, with staged responses interpreted by mothers as being indicative of anticipation, hunger, appreciation of food and satiation, as represented in Figure 1.

In this study, just seven of the 19 participants reported increased activity with maternal hunger, whilst 12 did not. When a theme is identified in a qualitative study it is important to consider the negative cases. When participants who reported increased fetal activity in association with maternal hunger were compared to those who did not it was noted that those women in the study who had provided the most complex and detailed descriptions of their baby’s movements were more likely to describe their babies as ‘very active’ or as ‘moving all the time’ recounted increased fetal activity with hunger. As the cohort went on to birth it was noted that babies born to mothers who described increased fetal movements with hunger were smaller than those that did not describe this pattern (3221.75 g vs. 3583.75 g) with a mean birthweight difference between groups of 364 gm. This difference was compared using a two tailed t test and did reach statistical significance (t(17) = -2.295, p = 0.035). Some analysis of other factors such as maternal BMI at booking, fetal sex and birth outcome was also conducted but is not reported here as no significant differences were identified in this small sample.

**Figure 2:**
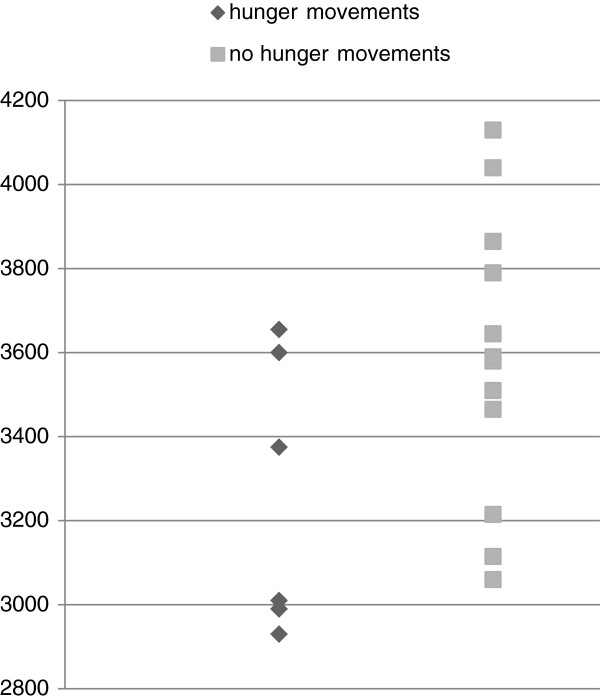
**Birthweight according to maternal report of increased fetal movements with hunger and no report of increased fetal movements with hunger.** Scatterplot of birthweight in grams of babies whose mothers reported increased fetal movement in the context of hunger (diamonds) and those who did not report increased fetal movement in the context of hunger (squares). Nb There are two babies of the same weight (2990 gm) in the hunger series.

**Figure 3:**
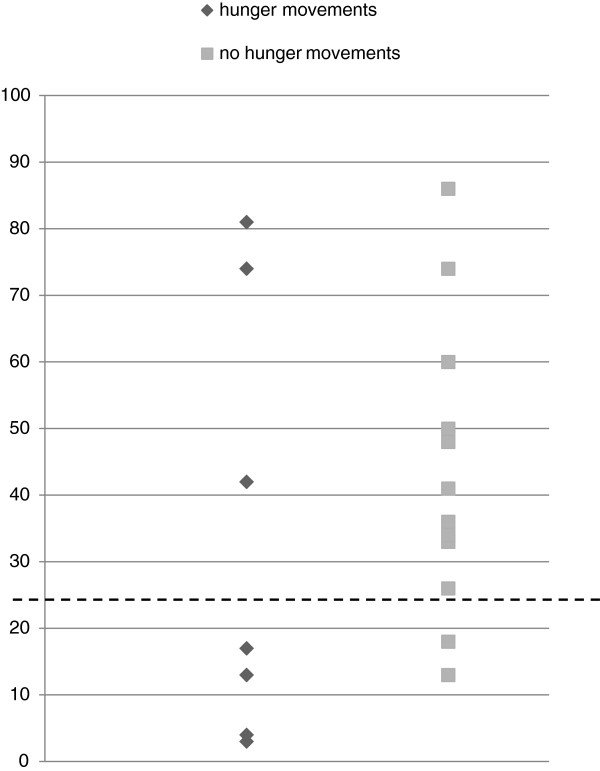
**Customised birthweight centile according to maternal report of increased fetal movements with hunger and no report of increased fetal movements with hunger.** Scatterplot of customised birthweight centile of babies whose mother reported increased fetal movements in the context of hunger (diamonds) compared to those who did not (squares). The 25^th^ centile is marked by a dashed line.

When infant birth weights are represented on a scatterplot as shown in Figure 2, it can be seen that there is a preponderance of smaller babies in the hunger group. When birthweights were converted to customised birthweight centile scores as shown in Figure 3 a similar distribution can be seen. Birthweight has a normal distribution, with the majority of newborns in any given sample expected to fall between the 25^th^ and 75^th^ centiles [25]. An association between patterns of fetal movement reported by pregnant women and fetal growth may help to explain why women frequently report concerning changes in fetal movements prior to stillbirth. However, this association must be interpreted with caution in a small sample such as this. In the quest to better understand normal and pathologic fetal activity, this study raises more questions than answers.

## Discussion

To the best of our knowledge human fetal behaviour commensurate with hunger and satiation has not previously been described in the literature. Anecdotal reports of increased fetal activity in association with meals are not new and a number of studies explored this possibility in the 1970s through until the early 1990s. The majority of such studies proposed that fetal activity was increased *following* a meal, failing to consider that the opposite may be true. Some investigators have concluded that increased fetal activity is seen with maternal glucose ingestion [26, 27], whilst others declared no such association [28–30]. At this time reviewers consider that there is either, no association between fetal activity and maternal meals [31] or that the issue is confounding and remains unresolved [32, 33].

Anecdotal reports of increased fetal movements with eating prompted the practice of offering women orange juice to stimulate fetal movement and expedite reactive cardiotocography (CTG) however a Cochrane review of this practice has concluded that there is no evidence that glucose or orange juice administered to the mother increases fetal movements or reduces non-reactive CTG [34].

In attempting to show that fetal movements increase with maternal glucose investigators have often reported the opposite. Fetal activity levels in relation to maternal blood sugar were evaluated in 10 diabetic women in the 36^th^ to 40^th^ week of pregnancy by Holden and colleagues [35]. Contrary to their hypothesis a statistically significant increase in fetal activity was seen during episodes of hypoglycaemia [35]. Another group investigated the effects of hyperglycaemia on the fetus in a group of nine healthy pregnant women when compared to six controls. Maternal blood glucose was maintained at set levels via intravenous infusion as per the glucose clamp technique developed by DeFranzo and colleagues [36]. A significant decrease in fetal movements was seen with sustained maternal hyperglycaemia [37].

A study of fetal cyclic motor activity in in relation to blood sugar level of mothers with Type 1 or gestational diabetes demonstrated that fetal cyclic motor activity became faster when maternal blood sugar levels decreased and slower when maternal blood sugar levels increased [38]. Devoe and colleagues [39] investigated the effects of maternal fasting and oral glucose on fetal biophysical parameters in 30 pregnant women at term. They reported that incidence of gross fetal body movements and fetal heart rate accelerations was lower following administration of 100 g of oral glucose when compared to fasting [39].

Patrick and colleagues [30] observed the gross fetal body movements of 31 pregnant women between 30 and 39 weeks gestation by continuous ultrasound recording over a 24 hour period. Women rested in a quiet room from 8 am to 8 pm during continuous ultrasound examination with interruptions for toilet breaks only. Meals were provided at set intervals and hourly maternal blood glucose measures were taken. Fetal activity levels were highest at the commencement of the recording when women were fasted and were seen to increase over the late evening, peaking at midnight, the same time period in which maternal blood glucose levels progressively dropped as the women went into their night time fast [30].

Controlled studies have demonstrated that administration of a glucose load to pregnant women results in an almost two-fold increase in fetal breathing movements [28, 40]. Fetal breathing movements are more often observed during a resting state, whilst gross fetal body movements occur more often in periods of apnoea [41]. Increased fetal breathing movements in association with higher maternal blood glucose levels, provides support in principle with the notion of gross fetal body movement quiescence post-prandially reported in this study.

Findings from these studies suggest an inverse relationship between maternal glucose and fetal activity, which is commensurate with the pattern of movements described by participants in the present study. Further the pattern of fetal movements in relation to meals described by women in this study is homologous to that established in animal studies in relation to feeding. In animal studies the period prior to meals is characterised by increased locomotor activity [42, 43]. Foraging animals may spend a considerable period of time actively procuring food in this phase [43, 44]. Following consumption of a meal, animals will generally become distracted from eating once they are sated and may spend a short period grooming or engaged in other activities before settling down for a sleep [43, 44]. The time period for this transition varies between individuals but is generally one of increased activity in the pre-prandial phase and rest in the post-prandial phase.

### Mood and food

Maternal descriptions of fetal activity at mealtimes in this study emphasised that fetal activity was indicative of fetal mood in relation to prandial state, especially ‘frustration’ at being hungry and ‘excitement’ or ‘contentment’ at being fed. It is not unreasonable that actions in relation to hunger and satiation be interpreted as emotions. The connection between feeding and the brain has been acknowledged since the Russian Physiologist Pavlov demonstrated anticipation of food by salivation in dogs exposed to the stimulus of a ringing bell [45]. Hormones involved in hunger and satiety have known central nervous system effects, including acting on parts of the brain controlling learning and emotion [46].

The empathy displayed in the participant’s appreciation of their baby’s perceived emotional state in relation to hunger and satiation might be considered an example of pre-birth formation of a mother-infant bond. Maternal-infant interaction and especially maternal responsiveness to infant cues, is essential to growth and development of infants after birth [47]. DiPietro (2010) asserts that the development of a mother-infant bond begins well before birth and is mediated by the hormonal milieu, with active inputs into the shaping of the intra-uterine environment coming from both the mother and the fetus, “while the uterus is the developmental niche of the fetus, it has become increasingly clear that the fetus is also an active inhabitant of that niche.” P 35 [48]. Maternal-fetal interaction around mealtimes could be considered an example of such a bond.

### Fetal food-seeking; an evolutionary adaptation?

For much of our existence as a species, humans have survived in environments of food scarcity. It is plausible that a fetal response to low energy-supply may have evolved as a mechanism for ensuring optimum availability of nutrients to maintain pregnancy. John Bowlby (1969) in his study of mother-infant bonding coined the term ‘Environment of Evolutionary Adaptedness’ (EEA), tracing many infant responses to the conditions for survival in our nomadic hunter-gather past [49]. In Bowlby’s theories of attachment, maternal and infant interests are often dovetailed [50]. Maternal receptiveness to a fetal mechanism for prompting eating where energy supplies are low, could arguably serve not just the interests of the growing fetus but also the pregnant woman, who optimally nourished will better withstand labour, birth and the subsequent demands of lactation.

### Fetal activity, fetal growth and stillbirth

In this study maternal reports of increased fetal activity in the context of hunger were associated with smaller size of the baby at birth. Both small and large fetal size is associated with increased risk of stillbirth [51] and the potential relationships between fetal activity and birthweight warrants further exploration. Animal studies demonstrate that in the context of placental insufficiency fetal metabolic changes such as increased insulin precede a drop-off in fetal growth [52]. In intra-uterine growth restricted (IUGR) pregnancies there is a larger maternal-fetal glucose concentration gradient, with the IUGR fetus being relatively hypoglycaemic [53]. It may be that fetal responses to maternal hunger and satiation were preferentially reported in this study by women who later delivered small babies as these fetuses were more sensitive to changes in maternal glucose due to metabolic adaptations to an environment of reduced energy supply via the placenta. A larger sample size would be needed to explore this hypothesis.

The relationship between IUGR, stillbirth and decreased fetal movements, is well documented [54, 55]. It has been hypothesised that decreased fetal movements might be a compensatory measure to reduce energy expenditure in the context of placental insufficiency [54]. The small infants in this study were well newborns, with normal Apgar scores who followed a normal neonatal course. Hypothetically, fetal food-seeking behaviour might be a compensatory mechanism employed by a healthy fetus in response to low fuel-supply. Decreased fetal movements in the context of IUGR or severe malnutrition might thus represent a de-compensation or loss of food-seeking response where energy supply has become severely diminished. In a study of 26 women who had experienced stillbirth, 22 reported a premonition that something had happened to their baby prior to diagnosis of fetal death. A key category emerging from interviews with those mothers was ‘losing contact with the baby’. Losing contact was frequently exemplified by mothers as absence of expected movements at mealtimes [10].

### Fetal movement patterns

Temporal fluctuations in fetal activity in this study were described by most participants (14/19) as being closely related to meals. Temporal aspects of fetal activity are of interest as a possible source of clues as to how fetal activity changes perceived by the mother might warn of impending stillbirth [56]. It has been established that human fetal activity has a complex temporal nature in that alternating bouts of activity and rest occur throughout the day and night with increased activity over the evening period and increased incidence of quiescent periods during the day [57]. Yet the mechanisms for controlling these fluctuations are not understood. The possibility that maternal meals may influence these fluctuations should be considered. The diurnal fetal activity pattern is periodically abolished following administration of steroids to pregnant women for fetal lung maturation [58], a response that may well be due to the disruption to maternal glucose regulation that occurs with this therapy.

Patterns of fetal activity are known to be altered in pregnancy pathologies which also involve alterations in energy supply to the fetus including IUGR and diabetes. Abnormalities in behavioural state cycling precede abnormalities in general movement quality in IUGR fetuses [59], whilst fetuses of diabetic women exhibit delayed behavioural state organisation [60] and changes in fetal behaviour are seen on ultrasound prior to evidence of ‘brain-sparing’ [61]. It has been hypothesised that these changes are indicative of neurodevelopmental delay in these fetuses, however they might just as likely be indicative of altered metabolic environment [62].

### Fetal quiescence postprandially

Reporting of fetal quiescence in the context of maternal postprandial state by some participants in this study raises the possibility that increased glucose supply might have a suppressive effect on fetal activity. Glucose metabolism requires consumption of oxygen. A study of glucose metabolism in fetal lambs demonstrated that fetal oxygen consumption is almost entirely accounted for in the metabolism of glucose supplied by the mother [53]. Oxygen requirements for glucose metabolism, either in the postprandial period or where maternal glucose is chronically elevated might therefore compete with oxygen requirements for movement in the fetus.

Decreased fetal movements are more frequently reported by overweight or obese pregnant women [6, 63]. Although the cause for this is unknown, it has been postulated that obese women have decreased sensitivity to fetal movements due to excess adipose tissue, reducing the impact of any fetal movement impulse on maternal skin [64]. The possibility of actual reduction in fetal movements in obese women during pregnancy, as opposed to reduced perception has not been ruled out. Studies have shown fetal activity is reduced in pregnancies complicated by diabetes [62, 65]. In diabetic pregnancies, resulting in fetal hyperglycaemia and hyperinsulinaemia, the risk of fetal hypoxemia and acidaemia are increased due to competing oxygen requirements [33]. Maternal obesity is known to be associated with increased blood glucose levels in pregnancy [66, 67]. Where the obese woman’s metabolic disturbances do not meet levels diagnostic of gestational diabetes, the fetus may nonetheless be exposed to an augmented glucose supply and an increased oxygen demand, with a potentially suppressive effect on movements.

In a study of 46 diabetic pregnant women a relationship between fetal activity levels and subsequent birthweight was established [68]. Glucose mediated macrosomia was seen exclusively amongst fetuses determined to be ‘inactive’ before birth. The authors postulated that the reason for the observed relationship between fetal activity and birthweight in babies of their participants may be an intrinsic tendency for the smaller babies in their study to be more active (the fidgety fetus hypothesis), which protected them from macrosomia [68]. However, they also demonstrated that higher birthweight of the infants in their study was correlated with higher maternal post-prandial blood glucose levels (*r* = 0.704). An alternative or supplementary explanation for their finding is that fetal activity levels provided a sensitive indicator of the glucose supply to fetuses in the study; a relationship borne out in the infant’s later birthweights.

## Conclusion

This study involved a small group of low-risk primiparous women from one provincial city in New Zealand and findings cannot therefore be applied to a broader pregnant population without further investigation of the hypotheses generated. The study was not designed to test any hypothesis but rather had an inductive or exploratory design, seeking to explore first-hand qualitative accounts of normal fetal activity by pregnant women. The finding of a fetal movement pattern in relation to meals thus emerged unsolicited. Larger carefully-planned studies would be needed to establish the nature of fetal responses to maternal hunger and eating including any potential implications in relation to fetal growth.

In this study increased fetal activity was commonly reported by participants to be associated with mealtimes. Fetal activity in association with maternal hunger in this study was preferentially reported by women who later delivered small infants, suggesting these movements are a compensatory response for low fuel-supply. The virtual discontinuation of research into human fetal responses to maternal blood glucose levels during the 1990s may have been premature. Further investigation of the fetal response to maternal meals as a complex multi-phase phenomenon is warranted given the potential for improving understanding of disorders of fetal growth and development of food-seeking behaviour before birth.
